# Supplementary material for: Kindlin-2 inhibits TNF/NF-κB-Caspase 8 pathway in hepatocytes to maintain liver development and function
Source: eLife. 2023 Jan 9;12:e81792. doi: 10.7554/eLife.81792 (PMC9848388; doi:10.7554/eLife.81792)
Supplement: Supplementary file 1. — (a) Antibody information. (b) Primer information for mouse. (c) primer information for human. [file elife-81792-supp1.docx]

**Supplementary file 1a: antibody information**

| **Name** | **Supplier** | **Cat no.** | **Application** |
| --- | --- | --- | --- |
| Kindlin-2 | Merck Millipore | MAB2617 | WB (1:1000) |
| Gapdh | ZSGB-BIO | TA-08 | WB (1:5000) |
| Bax | Cell Signaling technology | 5023S | WB (1:1000) |
| Bcl2 | Abcam | ab59348 | WB (1:1000) |
| Pcna | Abcam | Ab18197 | WB (1:1000) |
| Tubulin | Cell Signaling Technology | 2128 | WB (1:5000) |
| Cyclin D1 | Abcam | Ab16663 | WB (1:1000) |
| α-Sma | Absin | Abs120451 | WB (1:1000)  IHC (1:200) |
| Caspase3 | Sigma | MAB10753 | IHC (1:200) |
| Cleaved caspase3 | Cell Signaling Technology | 9661 | IHC (1:200) |
| F4/80 | Cell Signaling technology | 70076 | IHC (1:200) |
| Desmin | Cell Signaling technology | 5332 | IF (1:200) |
| Ck19 | Cell Signaling technology | 12434 | IHC (1:200) |
| Actin | ZSGB-BIO | TA-09 | WB (1:5000) |
| Ki67 | Cell Signaling technology | 12202 | IHC (1:200) |
| CD19 | Invitrogen | 13-0193-85 | IHC (1:200) |
| Ly6G | Santa | Sc53515 | IF(1:200) |

**Supplementary file 1b: primer information for mouse**

| Gene | Forward | Reverse |
| --- | --- | --- |
| Apoa | GGCACGTATGGCAGCAAGAT | TAGTCTCTGCCGCTGTCTTTGA |
| Ttr | CCATGAATTCGCGGATGTG | AGCCGTGGTGCTGTAGGAGTA |
| Fbn | GGATGGCAGCGTCGACTTT | TGGCAAGCCACAGTACTTCTTC |
| Tnfα | CCACGTCGTAGCAAACCACC | GATAGCAAATCGGCTGACGG |
| Col1a1 | TAGGCCATTGTGTATGCAGC | ACATGTTCAGCTTTGTGGACC |
| Col6a3 | ACTGGAACCACGGAAGTTCA | GTCACTTCCAACATCGAGGC |
| Tgfβ1 | GTGGAAATCAACGGGATCAG | ACTTCCAACCCAGGTCCTTC |
| Timp1 | AGGTGGTCTCGTTGATTTCT | GTAAGGCCTGTAGCTGTGCC |
| Acta2 | GTTCAGTGGTGCCTCTGTCA | ACTGGGACGACATGGAAAAG |
| Gs | TGGTCTGAAGTGCATTGAGGAG | CACCGGCAGAAAAGTCGTTG |
| Kindlin-2 | TGGACGGGATAAGGATGCCA | TGACATCGAGTTTTTCCACCAAC |
| Gapdh | TTTCTTCTTGCCTTGGGAGA | AGTTCCGCACTTCATTCAGG |

**Supplementary file 1c: primer information for human**

| BIRC3 | CTGGGCAGCAGGTTTACAA | GCATTCTTTGGATAGTAAAACACCA |
| --- | --- | --- |
| KINDLIN-2 | GACCATGGCGGACAGTTCTT | TTCTCGCTGTTATCTGCTTGT |
| GAPDH | TCGGAGTCAACGGATTTGGT | TTCCCGTTCTCAGCCTTGAC |
| TNFα | TAGCCCATGTTGTAGCAAACC | GCTCTTGATGGCAGAGAGGA |
